# Methylenetetrahydrofolate reductase (*MTHFR*) C677T polymorphism and high plasma homocysteine in chronic hepatitis C (CHC) infected patients from the Northeast of Brazil

**DOI:** 10.1186/1475-2891-10-86

**Published:** 2011-08-19

**Authors:** Erika RF Siqueira, Cláudia PMS Oliveira, Maria TC Muniz, Filipe Silva, Leila MMB Pereira, Flair J Carrilho

**Affiliations:** 1 Department of Gastroenterology LIM-07, University of Sao Paulo School of Medicine, Avenue Dr Arnaldo, 455, Sao Paulo, 01246903, Brazil; 2 Department of Gastroenterology, University of Pernambuco School of Medicine, Avenue Agamenon Magalhães, Pernambuco, 50.100-010, Brazil; 3 Departments of Biochemistry, University of Pernambuco School of Medicine, Avenue Agamenon Magalhães, Pernambuco, 50.100-010, Brazil; 4 Liver Institute of Pernambuco, Arnóbio Marques Street, 282, Pernambuco,50.100-130, Pernambuco, Brazil

**Keywords:** Hepatitis C, *MTHFR*, Genotype 1, Steatosis, Homocysteine

## Abstract

**Background/Aim:**

Hyperhomocysteinemia due to Methylenetetrahydrofolate Reductase (*MTHFR*) gene, in particular the C677T (Ala222Val) polymorphism were recently associated to steatosis and fibrosis. We analyzed the frequency of *MTHFR *gene in a cross-sectional study of patients affected by Chronic Hepatitis C (CHC) from Northeast of Brazil.

**Method:**

One hundred seven-four untreated patients with CHC were genotyped for the C677T *MTHFR*. Genomic DNA was extracted from peripheral blood cells and the C677T *MTHFR *polymorphism was identified by PCR-RFLP. The homocysteine (Hcy) levels were determined by chemiluminescence method. All patients were negative for markers of Wilson's disease, hemochromatosis and autoimmune diseases and have current and past daily alcohol intake less than 100 g/week.

**Results:**

Among subjects infected with CHC genotype non-1 the frequency of *MTHFR *genotypes TT was 9.8% *versus *4.4% genotype 1 (p = 0.01). Nevertheless, association was found between the *MTHFR *genotype TT × CT/CC polymorphism and the degree of steatosis and fibrosis in both hepatitis C genotype (p < 0.05). A significant difference was found on plasma Hcy levels in patients with steatosis regardless of HCV genotype (p = 0.03).

**Conclusion:**

Our results indicate that plasma Hcy levels is highly prevalent in subjects with chronic hepatits C with steatosis regardless of HCV genotype and vitamin deficiency. The presence of genotype TT of *MTHFR *C677T polymorphism was more common in CHC genotype non-1 infected patient regardless of histopathological classification and genotype TT+CT frequencies were significant in the presence of fibrosis grade 1+2 and of steatosis in CHC infected patients from the northeast of Brazil regardless of HCV genotype. The genetic susceptibility of *MTHFR *C677T polymorphism should be confirmed in a large population.

## Introduction

Homocysteine (Hcy) belongs to a group of molecules known as cellular thiols. It is considered a "bad thiol" because its association with a variety of health conditions including cardiovascular disease, [1] end-stage renal disease, [2] neural tube defects, [3] and cognitive dysfunctions including Alzheimer disease [4]. Recently, homocysteine has also been implicated in the pathogenesis of alcoholic liver injury [5].

One of the most common mutations, or polymorphisms, that are associated with a mild increase in plasma homocysteine (hyperhomocysteinemia) is the 677C→T substitution (an alanine to valine change) in the enzyme methylenetetrahydrofolate reductase (MTHFR). The MTHFR is an enzyme of the folate metabolism that reduces 5,10- metilenetetraidrofolate (5,10-mTHFR) to 5-metiltetraidrofolate (5-mTHF), an important co-factor to homocysteine (Hcy) methylation. Mutations in *MTHFR *gene like C677T result in amino acids substitutions that lead to a decreased enzyme activity [6,7]. As a consequence of the *MTHFR *dysfunctions, an increased Hcy level in plasma has been expected which, in turn, produces a cytotoxic effect [8].

The frequency of this variant in the homozygous state varies from 0% to1% in African Americans to 25% in Hispanic Americans, ranging for most populations (Canada and United States, Europe, Asia, and Australia) between 10% to 15% [9]. Recently, it has been shown that hyperomocysteinaemia, in relationship with the methylenetetrahydrofolate reductase, *MTHFR *C677T polymorphism, favours steatosis and fibrosis in HCV infected immune competent individuals through an alteration of lipid metabolism [10].

Human hepatitis C virus (HCV) infects about 2-3% of the world population. HCV infection leads to chronic hepatitis in up to 60-80% of infected individuals [11] and is associated with liver steatosis, fibrosis, cirrhosis, and hepatocellular carcinoma (HCC) [12].

Most studies have reported approximately 50% prevalence of steatosis among patients undergoing a liver biopsy because of HCV [13,14]. In patients with HCV infection there is a ''metabolic fat'' (especially in patients with HCV genotype 1) and a ''viral fat'' (especially in patients with genotype 3).

Genotype 3 is the only subtype that has been shown to correlate with a higher grade of steatosis independent of other host-related factors, such as the presence of nonalcoholic fatty liver disease (NAFLD) [15]. The severity of steatosis in these patients is directly related to the burden of the HCV RNA viral load, and resolution of steatosis is often observed with the loss of viremia after antiviral treatment [16,17]. It has been postulated that HCV genotype 3 can cause steatosis also by interfering with triglyceride secretion. Otherwise, in genotype 1 infection is attributable to metabolic perturbations caused by activation of proinflammatory mechanisms and underlying obesity and insulin resistance.

The aim of the present study was to investigate whether *MTHFR *C677T polymorphism might play a role in progression of fibrosis and steatosis in hepatitis C patients from Northeast of Brazil and correlate with homocysteine levels according to histological grades of fibrosis and steatosis.

## Patients and methods

### Patients

We studied one hundred seven-four naive patients with chronic hepatitis C infection (CHC) from the Northeast of Brazil (91 male, 83 female). All patients enrolled had increased aminotransferase levels for at least six months and tested positive for anti-HCV antibodies (third-generation enzyme immunoassay) and HCV-RNA (RT-PCR, Roche Cobas Amplicor 2.0, Roche Diagnostics, Basel, Switzerland).

The HCV genotype, determined by LiPA assay (Innolipa HCV II; Immunogenetics, Ghent, Belgium). All patients were enrolled at the Liver Institute of Pernambuco in Brazil from February 2007 to October 2009.

This cross-sectional study was conducted according with the Helsinki declaration of 1975. Specific informed consent was obtained for the study and the protocol was approved by the Internal Review Board of the University of Pernambuco- Brazil. The investigations performed to exclude other causes of liver disease included a hepatobiliary system ultrasound, viral serology, autoantibody titers, serum iron, ferritin and transferrin saturation, ceruloplasmin and copper levels, and alpha1-antitrypsin. Patients who had a > 100 g/week alcohol intake determined by a detailed personal history, questioning of family members, and an investigation of previous medical records, were excluded. Also it was excluded treatment with immunosuppressive drugs or drugs causing steatosis (corticosteroids, antiepileptic agents, tamoxifen and amiodarone).

The diagnosis of diabetes type II, hypertension, and dyslipidemia were based on the criteria of the American Diabetes Association (Alexandria, VA, USA); fasting glucose ≥ 100 mg/dL; triglyceride (Tg) ≥ 150 mg/dL; high density lipoprotein (HDL) < 40 mg/dL in men or < 50 mg/dL in women; and ≥ 130 mmHg systolic or ≥ 85 mmHg diastolic) [18]. The folic acid and B12 vitamin reference were 3.1-17.5 ng/mL and 197-866 pg/mL respectively.

Normative reference Hcy levels were considered to be 12 or less (μmol/L) in males and 10 or less in females [10]. The homocysteine cut-off level in this study was 9 μmol/L determined by ROC curve.

### Study Design and Laboratory assays

The *MTHFR *polymorphism was analyzed in all 174 patients, however only in 138 of these patients the serum samples were collected at the time of liver biopsy. Thus, we used 138 serum samples to determine total cholesterol, low-density lipoprotein (LDL), high-density lipoprotein (HDL), triglycerides (Tg), alanine aminotransferase (ALT), aspartate aminotransferase (AST), gamma-glutamyl transferase (γGT), alkaline phosphatase (AP), fasting glucose, fasting insulin, and insulin resistance (homeostasis model assessment-insulin resistance [HOMA-IR]: fasting insulin (U/mL) fasting glucose (mmol/L)/22.5) [19]. For insulin resistance the cut-off value was considered to be ≥ 2.5. Blood samples were centrifuged within 60 min to separate plasma, serum and leukocyte cells and storaged at-80 °C.

The homocysteine levels were determined by chemiluminescence method [20]; Fasting Glucose, total cholesterol and fractions, triglycerides, ALT, AST, AP, γGT and fasting insulin were performed by standard methods using automated techniques (Cobas, Roche). The LDL cholesterol was determined by Friedwald equation [21].

The C677T polymorphism was determined by a polymerase chain reaction restriction fragment length polymorphism (PCR-RFLP) assay. The C→T transition creates a restriction site for the enzyme *Hinf *I and the digested product was isolated electrophoretically in 2% agarose gel and the fragments were visualized in ultraviolet light (UV) after being stained with ethidium bromide. Wild type (CC) shows a single fragment of 198 bp; heterozygote (CT) shows fragments of 198, 175 and 23 bp; and mutant homozygote (TT) shows two fragments with 175 and 23 bp [22].

### Histological analysis

The liver tissue was fixed in 4% formaldehyde and processed for hematoxylin-eosin and Masson trichrome stains for histological analysis. Histological analyses were evaluated by only one pathologist who was unaware of the HCV genotype and of the patient's clinical characteristics. Stages of fibrosis and grades of inflammation were scored according to METAVIR, that it consists of F0 (no fibrosis), F1 (portal fibrosis without septa), F2 (portal fibrosis with few septa), F3 (numerous septa without cirrhosis), F4 (cirrhosis). Steatosis was graded 0-3 based on percentages of hepatocytes harbouring lipid droplets in the biopsy (0 reflecting none; 1 equalling 40-33%; 2 referring to 33-66%; and 3 representing > 66% steatotic hepatocytes).

### Statistical analysis

Data analysis was performed with SPSS 15.0 software. Distribution normality of the groups considered was preliminary evaluated by Kolmogorov-Sminorv test. Differences between groups were analyzed by analysis of variance (ANOVA) when variables were normally distributed. Chisquare test or Fisher's exact test were used to compare categorical variables. Logistic regression analysis was used to identify independent predictors for *MTHFR *polymorphism, gender, triglyceride, fibrosis and steatosis. The proportion of *MTHFR *alleles were distributed in patients in accordance with the Hardy-Weinberg equilibrium. Results were considered significant when the *p *value was less than 0.05.

## Results

### Clinical and biochemical analysis

In the present study 174 patients with CHC were included. There were 52.3% (91/174) males and 47.7% (83/174) females. The biochemical characteristics according genotype and histological classification were only analyzed in 138 patients with CHC and the patients were stratified according to viral genotype 1 (n = 93) and non-1 (n = 45).

The biochemical characteristics according to the genotype classification demonstrated Hcy levels and concentrations of total cholesterol differ significantly between patients with genotype 1 and genotype non-1 (9.96 *versus *9.39 μmol/L and 158.01 *versus *138.58 mg/dL, respectively, p = 0.01) (Table 1). The Hcy level differs significantly between no steatosis and steatosis (9.0 *versus *10.3 μmol/L respectively, p = 0.03) (Table 2). Although neither folate and vitamin B12 nor triglycerides, total cholesterol, HDL, LDL, HOMA, glucose and Hcy level differ between genotypes frequencies of the 677C/T (*MTHFR*) polymorphism (p > 0.05) (Table 3).

**Table 1:** Clinical and biochemical characteristics of CHC virus infection patients according genotype classification

	*GENOTYPE 1* *(N = 93)*	*GENOTYPE NON-1* *(N = 45)*	P
Age	54.06	51.59	0.14
Fasting Glucose	92.58	100.13	0.80
Homocysteine* (μmol/L)	9.96	9.39	0.01*
HOMA	2.72	3.35	0.74
AST (UI)	68.69	73.24	0.97
ALT(UI)	84.02	91.56	0.58
γGT(UI)	86.06	84.31	0.93
AP(UI)	83.03	72.67	0.26
Total Cholesterol*(mg/dL)	158.01	138.58	0.01*
HDL (mg/dL)	49.05	46.73	0.29
LDL(mg/dL)	88.00	74.44	0.10
**Triglycerides (mg/dL)**	105.89	91.69	0.05*

*p significative

References values:

AST: Male- 10-34 UI, Female- 10-36 UI; ALT: Male- 10-44 UI, Female-10-36 UI;

γGT: Male- 11-50 UI, Female-7-32 UI; AP: Male- 45-122 UI, Female-32-104 UI; Cholesterol: < 200

mg/dl; Triglycerides: < 150 mg/dl; HDL: > 40 mg/dl; Glucose: < 100 mg/dl; HOMA homeostasis

model assessment for insulin resistence (value < 2.5), Homocysteine < 9.0 μmol/L.

**Table 2:** Plasma levels of Homocysteine in CHC virus infection patients according genotype and histopathological classification

	*Homocysteine* *Media ± SD *	p
**Fibrosis 1+2 (n = 105)**	9.7 ± 4.7	
**Fibrosis 3+4 (n = 40)**	10.4 ± 2.9	0.30
**No Steatosis (n = 46)**	9.0 ± 2.8	
**Steatosis (n = 99)**	10.3 ± 4.8	0.03*
**Genotype 1 (n = 96)**	10.1 ± 2.9	
**Genotype Non -1 (n = 49)**	9.5 ± 6.1	0.55

References values: Homocysteine < 9.0 μmol/L *p significative

**Table 3:** Biochemical characteristics of the 677C/T (*MTHFR*) polymorphisms in CHC

*Variables*	*CT+TT* *Media ± SD (n = 65)*	*CC* *Media ± SD (n = 73)*	*p*
**Total Cholesterol**	147.3 ± 33.7	155.6 ± 43.8	0.21
**HDL**	47.5 ± 14.4	49.0 ± 13.9	0.55
**LDL**	79.7 ± 30.4	87.0 ± 39.8	0.23
**Triglyceride**	103.4 ± 40.6	99.4 ± 45.9	0.59
**Fasting Glucose**	97.4 ± 37.5	93.0 ± 18.1	0.39
**Folate**	12.9 ± 2.8	12.4 ± 2.8	0.33
**Vit.B12**	766.7 ± 361.8	652.8 ± 278.3	0.09
**HOMA**	3.2 ± 2.9	2.7 ± 1.8	0.21
**Homocysteine**	10.2 ± 5.4	9.5 ± 2.8	0.31

References values

Triglyceride < 150 mg/dL, Total Cholesterol < 200 mg/dL, LDL-c < 130 mg/dL, HDL-c > 40 mg/dL, HOMA: < 2.5.

Glucose: < 100 mg/dl, Folate 3.1-17.5 ng/mL, Vit. B12 197-866 pg/mL, Homocysteine < 9.0 μmol/L.

### *MTHFR *677C/T polymorphism

The *MTHFR *polymorphism was analyzed from peripheral blood of 174 patients. The genotype TT was more frequent in HCV non-1 genotype than genotype 1 (9.8% *versus *4.4% respectively, p = 0.01) (Table 4). Associated with this no relation was observed in the genotype frequencies of the 677C/T (*MTHFR*) polymorphism according to HCV genotype and histopathological classification (p > 0.05) (Table 5). Hence, a significant difference was observed in the genotype TT+CT frequencies according to histological grades of fibrosis (1+2 [n = 116] *versus *3+4 [n = 58]) (p = 0.001) and of steatosis (No Steatosis [n = 70] *versus *Steatosis [n = 104]) (p = 0.04) regardless of HCV genotype (Table 6).

**Table 4:** Genotype frequencies of the 677C/T (*MTHFR*) polymorphisms in CHC patients according genotype and histopathological classification

	*Genotype frequencies (%)*			
*MTHFR*	TT	CT	CC	p
**Steatosis (n = 104)**	5.7	40.4	53.8	0.80
**No Steatosis (n = 70)**	7.1	41.4	51.4	
**Fibrosis 1+2 (n = 116)**	5.1	37.9	56.9	0.21
**Fibrosis 3+4 (n = 58)**	8.6	46.5	44.8	
**Genotype 1 (n = 113)**	4.4	35.4	60.1	0.01*
**Genotype Non-1 (n = 61)**	9.8	50.8	39.3	

*p significative

**Table 5:** Genotype frequencies of the 677C/T (*MTHFR*) polymorphisms in CHC patients according genotype and histopathological classification

*MTHFR*	*Genotype 1* *Genotype frequencies (%)*		*p*	*Genotype non 1* *Genotype frequencies (%)*		*p*
	CT+TT	CC		CT+TT	CC	
**Fibrosis 1+2**	37 (48.7%)	39 (51.3%)	0.43	19 (47.5%)	21 (52.5%)	0.80
**Fibrosis 3+4**	15 (40.5%)	22 (59.5%)		11 (52.4%)	10 (47.6%)	
**No Steatosis**	23 (48.9%)	24 (51.1%)	0.70	08 (34.8%)	15 (65.2%)	0.11
**Steatosis**	29 (43.9%)	37 (56.1%)		22 (57.9%)	16 (42.1%)	

**Table 6:** Genotype frequencies of the 677C/T (*MTHFR*) polymorphisms in CHC patients according to histopathological classification

	*MTHFR* *TT+CT (%)*	*p*
Fibrosis 1+2 (n = 116)	68.3	
Fibrosis 3+4 (n = 58)	31.7	0.001*
No Steatosis (n = 70)	37.8	
Steatosis (n = 104)	62.8	0.04*

*p significative

In multi regression analysis no relation were observed among MTHFR polymorphism, Hcy level, HCV genotype and lipid profile as a independent variables for steatosis and fibrosis (Table 7 and 8).

**Table 7:** Multi regression analysis in which *MTHFR *polymorphism, Homocysteine level, HCV genotype and lipid profile as a independent variables for steatosis

*Variables*	*OR (CI 95%)*	p*	*OR adjusted*	*p*	*OR adjusted*	*p*
Gender: F/M	1.1 (0.6-2.0)	0.88	1.1(0.5-2.3)	0.90	1.1 (0.5-2.3)	0.87
HOMA ≥ 2.5	1.5 (0.7-3.2)	0.35	1.3 (0.6-2.9)	0.40	1.4 (0.7-3.1)	0.35
LDL cholesterol ≥ 130	0.5 (0.2-1.6)	0.35	0.6 (0.2-1.9)	0.35	- (**)	-
HDL cholesterol ≤ 40	1.1 (0.5-2.4)	0.84	0.9 (0.4-2.1)	0.82	1.0 (0.4-2.2)	0.93
Total Cholesterol ≥ 200	0.8 (0.2-2.9)	0.75	- (**)	-	0.9 (0.2-3.6)	0.96
Triglyceride ≥ 150	0.6 (0.2-1.8)	0.38	0.6 (0.2-1.9)	0.40	0.6 (0.2-1.9)	0.37
TT+CT (*MTHFR*)	0.8 (0.4-1.5)	0.64	1.0 (0.5-2.1)	0.94	1.0 (0.5-2.1)	0.95
Homocysteine ≥ 9	1.6 (0.8-3.3)	0.18	1.6 (0.7-3.3)	0.24	1.6 (0.7-3.3)	0.25

*Fisher Test ** Collinearity

References values:

Triglyceride < 150 mg/dL, Total Cholesterol < 200 mg/dL, LDL-c < 130 mg/dL, HDL-c > 40 mg/dL, HOMA: < 2.5, Homocysteine < 9.0 μmol/L

CI: confidence interval

**Table 8:** Multi regression analysis in which MTHFR polymorphism, Homocysteine level, HCV genotype and lipid profile as a independent variables for fibrosis1+2

*Variables*	*OR (CI 95%)*	p*	*OR adjusted*	*p*	*OR adjusted*	*p*
Gender: F/M	0.9 (0.5-1.7)	0.87	1.1 (0.5-2.4)	0.85	1.1 (0.5-2.4)	0.82
HOM- ≥ 2.5	0.8 (0.4-1.8)	0.55	0.8 (0.4-1.8)	0.63	0.8 (0.4-1.8)	0.57
LDL cholesterol ≥ 130	2.1 (0.4-10)	0.51	2.2 (0.4-11.1)	0.34	-(**)	-
HDL cholesterol ≤ 40	1.0 (0.4-2.3)	1.00	1.3 (0.6-3.2)	0.51	1.3 (0.5-3.2)	0.53
Tot-l Cholesterol ≥ 200	1.7 (0.3-8.3)	0.73	-(**)	-	1.8 (0.3-9.2)	0.49
Triglyceride ≥ 150	1.4 (0.4-5.3)	0.76	1.3 (0.3-5.0)	0.70	1.3 (0.3-5.1)	0.72
TT+CT (*MTHFR*)	0.9 (0.5-1.7)	0.75	1.4 (0.7-3.1)	0.39	1.4 (0.6-3.1)	0.39
Homocysteine ≥ 9	0.6 (0.3-1.3)	0.17	0.7(0.3-1.5)	0.34	0.7 (0.3-1.5)	0.35

*Fisher Test ** Collinearity

References values:

Triglyceride < 150 mg/dL, Total Cholesterol < 200 mg/dL, LDL-c < 130 mg/dL, HDL-c > 40 mg/dL, HOMA: < 2.5, Homocysteine < 9.0 μmol/L.

CI: confidence interval

### Discussion

The heterogeneity of Brazilian population regarding racial definition mixed with social economic factors may represent a confounding factor herein. The absence of information on the reported genetic risk factors in the Northeast of Brazil population, which is considered to be genetically very heterogeneous, led us to design the present study. In our data we reported that the genotype TT was more frequent in the HCV genotype non-1 without association with histological grades of fibrosis and of steatosis. We also observed significant difference in the genotype TT+CT frequencies according to histological grades of fibrosis and steatosis regardless of HCV genotype.

Several biological and clinical implications have been suggested to occur in relationship with the *MTHFR *677C/T polymorphism. The *MTHFR *polymorphisms were found to be associated with increased cardiovascular risk in several populations including Lebanese, Japanese and French Canadians [23-25]. Toniutto et al., also found a relation between *MTHFR *677C/T polymorphism and liver fibrosis in patients who underwent liver transplantation with recurrent hepatitis C and also speculates that the *MTHFR *polymorphism could play a direct profibrogenic effect, modulating the action of proteins involved in collagen degradation [26].

Otherwise Borgia et al. did not find association with polymorphisms of *MTHFR *in the outcome of pegylated-IFNα plus ribavirin treatment in patients with chronic hepatitis C, only the homocysteine levels [27]. Silva et al., only confirms the association between increased plasma homocysteine concentration in Alzheimer's disease and suggests that C677T *MTHFR *polymorphisms not contributed to genetic susceptibility for Alzheimer's disease in elderly individuals in the Northeast of Brazil [28].

However, another study conducted in Northeast of Brazil, Couto et al., screened 843 neonates for *MTHFR *C677T polymorphism. The T677 allele frequency and TT677 genotype was higher than those observed in other studies of African-descent populations. The T allele frequency was 0.23 and the C/T and T/T genotypes prevalence were 36.2 and 5.3 percent, respectively [29].

The present study provides evidence that a genetic background, such as the *MTHFR *polymorphism through hyperhomocysteinemia induced derangement of lipid metabolism, may contribute to the development of higher degrees of steatosis, which in turn accelerates the progression of liver fibrosis in chronic HCV infection. Potential mechanisms of this effect may include the increased sensitivity of steatotic livers to oxidative stress, cytokine-mediated injury, and steatosis-related hepatic insulin resistance [30].

Other important finding of our study was the higher Hcy level in patients with steatosis, although the *MTHFR *polymorphism was not identified as a risk factor for steatosis in the whole population (HCV genotypes 1+ non-1) in the multi regression analysis. It should be understood because the high plasma levels of Hcy have been reported to negatively influence normal cell function in many different tissues, such as vascular endothelium and smooth muscle cells and the liver. These effects, in turn, may explain the association of hyperhomocysteinaemia with vascular disease (thrombosis of arterial and venous districts and atherosclerosis) [31-36] and more recently, have been advocated for a possible role in pathogenesis and evolution of chronic liver disease [36,37].

Hcy is a toxic non-protein sulfur containing amino acids in humans. It is formed exclusively upon demethylation of the essential amino acid- methionine. The Hcy metabolism occurs through the junction of the remethylation and transsulfuration pathways. These pathways are strongly influenced by enzymes polymorphisms. *MTHFR *enzyme has fundamental importance in plasmatic Hcy regulation [38,39].

Hcy decreases the expression of a wide range of antioxidant enzymes [40-42] and impairs endothelial nitric oxide (NO) bioavailability by inhibiting glutathione peroxidase activity raise the possibility that Hcy sensitizes cells to the cytotoxic effects of agents or conditions known to generate reactive oxygen species (ROS). Decreased NO bioavailability has also been shown *in vitro *to increase the expression of monocyte chemoattractant protein 1 (MCP-1) which may enhance intravascular monocyte recruitment and lead to accelerated lesion formation [43].

There is evidence that Hcy induced endoplasmic reticulum (ER) stress causes dysregulation of the endogenous sterol response pathway, thereby leading to increased hepatic biosynthesis and uptake of cholesterol and triglycerides without impairing the hepatic export of lipids [44]. Similar result was observed in Adinolfi et al., studies that investigated the role of these factors in the development of HCV related steatosis and in the progression of chronic hepatitis C in 116 patients, 50% had a body mass index (BMI) of 25 or higher; 58% were infected with HCV genotype 1, and 65.5% showed steatosis. According to multivariate analysis, steatosis was independently associated with hyperhomocysteinemia (OR = 7.1) [10].

We also observed lower concentrations of serum total cholesterol in CHC patients genotype non-1. Similar results have been described by Corey at al., who demonstrated that serum lipids play a role in hepatitis C virion circulation and hepatocyte entry. In a cohort of 179 patients with CHC this author showed that patients with HCV had lower concentrations of total cholesterol than in the control group. These data support the hypothesis that the lipo-viral particles use the LDL-C receptors of hepatocytes as points of entry of the virus. Once inside into hepatocyte, the replication dependents of the lipid environment of the host [45-47].

In summary, our study had important implications. According to our data, Hcy levels were highly prevalent in subjects with chronic hepatits C with steatosis regardless of HCV genotype and vitamin deficiency. The presence of genotype TT of *MTHFR *C677T polymorphism was more common in CHC genotype non-1 infected patient regardless of histopathological classification and genotype TT+CT frequencies were significant in the presence of fibrosis grade 1+2 and of steatosis in CHC infected patients from the northeast of Brazil. The genetic susceptibility of *MTHFR *C677T polymorphism should be confirmed in a large population.

## Conclusion

The *MTHFR *C677T polymorphism frequencies were significant in the presence of fibrosis and of steatosis in CHC infected patients from the northeast of Brazil regardless of homocysteine levels and HCV genotype.

## List of abbreviations

**ALT: **alanine aminotransferase; **AP: **alkaline phosphatase; **AST: **aspartate aminotransferase; **BMI**: body mass index; **CHC: **Chronic Hepatitis C; **ER: **endoplasmic reticulum; **GGT: **gamma-glutamyl transferase; **HCC: **hepatocellular carcinoma; **HCV: **human hepatitis C virus; **HCY: **homocysteine; **HDL: **high-density lipoprotein; **HOMA-IR: **homeostasis model assessment-insulin resistance; **LDL: **low-density lipoprotein; **MCP-1: **monocyte chemoattractant protein 1; **MTHFR: **methylenetetrahydrofolate reductase; **NAFLD: **nonalcoholic fatty liver disease; **NO: **nitric oxide; **PCR-RFLD: **polymerase chain reaction restriction fragment length polymorphism; **ROS**: reactive oxygen species; **5,10-mTHFR: **5,10: metilenetetraidrofolate; **5-mTHFR: **5-metiltetraidrofolate; **Tg**: triglyceride.

## Competing interests

The authors declare that they have no competing interests.

## Authors' contributions

ERFS- participated in the all steps of study, including design of the study, performed the statistical analysis and wrote the manuscript.

CPMSO- critical revision of the manuscript for important intellectual content.

MTCM-carried out the molecular genetic studies, participated in the sequence alignment and drafted the manuscript.

FSS- acquisition of data; analysis and interpretation of data.

LMMBP- conceived of the study, and participated in its design and coordination and helped to draft the manuscript.

FJC-study supervision

All authors read and approved the final manuscript.
